# microRNAs as Regulators of the Immune Response and Their Potential Therapeutic Applications in Cancer

**DOI:** 10.3390/ncrna12050034

**Published:** 2026-09-01

**Authors:** Ezgi Biltekin, Bulent Ozpolat

**Affiliations:** 1 Department of Nanomedicine, Houston Methodist Research Institute, Houston, TX 77030, USA; 2 Stephenson School of Biomedical Engineering, Stephenson Cancer Center, The University of Oklahoma, Norman, OK 73019, USA

**Keywords:** miRNA, cancer immunity, immune checkpoint blockade, PD-L1, PD-1, CTLA-4, immunotherapy, spatial transcriptomics, chemoresistance, targeted therapy

## Abstract

MicroRNAs (miRNAs) are small non-coding RNAs that have emerged as critical regulators of gene expression and key cellular processes in cancer, including tumor cell proliferation, survival, metastasis, and both innate and adaptive immune responses within the tumor microenvironment. Through their ability to modulate immune cell development, differentiation, polarization, antigen presentation, cytokine signaling and immune checkpoint expression, miRNAs play a vital role in shaping immune responses and represent attractive therapeutic candidates for remodeling the tumor immune microenvironment. This review provides a comprehensive overview of the mechanisms through which miRNAs regulate anti-tumor immunity and contribute to immune evasion with particular emphasis on their role in modulating major immune checkpoint pathways, including PD-1/PD-L1, CTLA-4, LAG-3, TIM-3 and CD28-mediated signaling. We further examine the contribution of miRNAs to therapeutic resistance and discuss their potential integration with immune checkpoint blockade, chemotherapy, PARP inhibitors and targeted therapies to enhance antitumor efficacy and overcome treatment resistance. Recent advances in spatial transcriptomics and computational approaches have expanded our understanding of miRNA-mediated regulatory networks at single-cell and tissue levels and provide new insights into tumor–immune interactions. In addition, we evaluate the current progress in the clinical development of miRNA-based therapeutics, including miRNA mimics and inhibitors and discuss the major challenges associated with efficient delivery, off-target effects, immunogenicity and patient heterogeneity. Collectively, the evidence highlights the growing potential of miRNAs as diagnostic biomarkers, therapeutic targets and therapeutic tools in cancer immunotherapy. Continued advances in RNA therapeutics, delivery technologies and multi-omics approaches are expected to accelerate the clinical translation of miRNA-based strategies for personalized cancer therapy.

## 1. Introduction

MicroRNAs (miRNAs) are small single-stranded non-coding RNAs, approximately 22 nucleotides in length, and have emerged as key post-transcriptional regulators of gene expression since their discovery in *Caenorhabditis elegans*. This groundbreaking discovery earned Victor Ambros and Gary Ruvkun the 2024 Nobel Prize in Physiology or Medicine [1]. Studies over the last three decades have demonstrated that miRNAs orchestrate diverse cellular processes and function as critical regulators of cell proliferation, differentiation, apoptosis, metabolism, oncogenic signaling pathways, and tumor progression [2].

**Figure 1:**
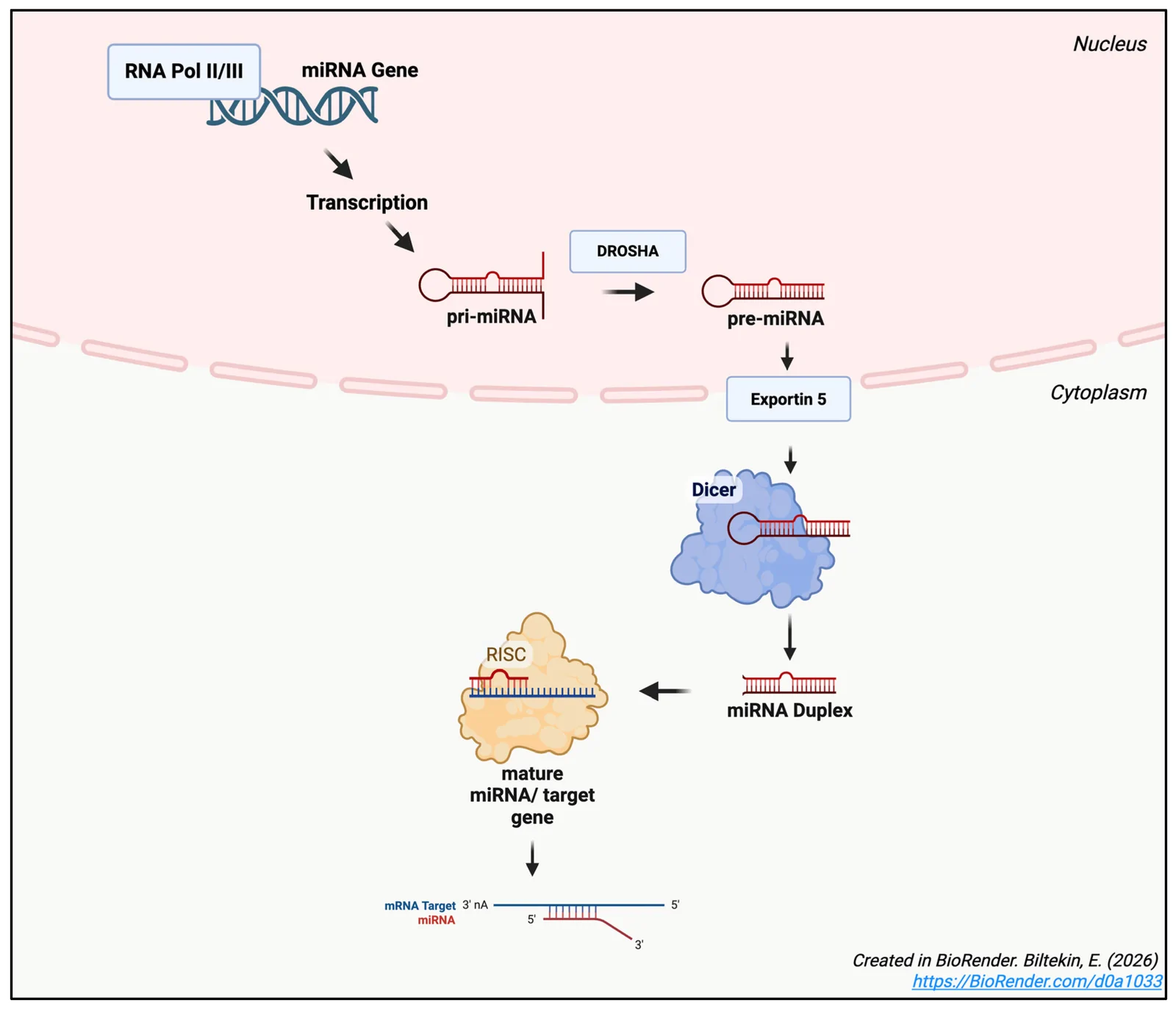
Schematic representation of miRNA biogenesis. Pri-miRNAs are processed by DROSHA and Dicer into mature miRNAs that are loaded into the RISC to regulate target mRNAs. Created with BioRender. Biltekin, E. (2026) https://BioRender.com/d0a1033 (accessed on 29 June 2026).

miRNAs are transcribed in the nucleus as primary miRNA (pri-miRNA) transcripts by RNA polymerase II or III, then processed into precursor miRNA (pre-miRNA) by the enzyme DROSHA and exported to the cytoplasmic compartment of the cell through the Ran-GTP/Exportin-5 nuclear transport complex proteins. Precursor miRNAs are processed by Dicer into miRNA duplexes—a double-stranded complex—which are later converted into a single-stranded mature miRNA that is capable of binding to either the 3′ untranslated region (3′UTR) or the open reading frame of target mRNAs, thereby silencing gene expression [3,4] (Figure 1).

The significance of miRNAs in oncogenesis was first recognized approximately 24 years ago with the identification of the downregulation/deletion of the miR-15a–miR-16-1 cluster in B-cell chronic lymphocytic leukemia (B-CLL) [5,6], which established miRNAs as bona fide tumor suppressors in human cancer. Their tumor-suppressive functions and roles in pro-oncogenic processes make miRNAs valuable candidates for diagnostic applications and the development of novel targeted therapeutic strategies [2].

During the multistep process of carcinogenesis, the immune system plays a complex role, acting either as a tumor-suppressive defense mechanism or as a promoter of cancer progression. Components of both innate and adaptive immunity may detect and eradicate tumor cells; however, chronic inflammation and the abundance of regulatory T cells (Tregs) and tumor-associated macrophages (TAMs) may instead promote oncogenesis [7]. In addition, tumor cells may undergo changes in their immunogenic profile that facilitate immune evasion. The discovery of immune checkpoint molecules, particularly CTLA-4 and PD-1/PD-L1, by Drs James Allison and Tasuku Honjo, who were awarded the 2018 Nobel Prize in Physiology or Medicine, revolutionized cancer immunotherapy by improving our understanding of tumor-immune cell interactions within the tumor microenvironment [8,9,10].

In this review, we focus on the miRNA-mediated regulation of the immune system and the crosstalk between tumor and immune cells, with particular emphasis on their therapeutic applications in cancer with the aim of addressing the central question of “Are miRNAs key players in regulating the immune response in tumorigenesis and what is their therapeutic potential in development of novel cancer therapy approaches?”.

## 2. MicroRNAs in Immune Regulation and Cancer Crosstalk

### 2.1. Regulation of Innate Immune Response by miRNAs

#### 2.1.1. Regulation of Effector Cells

Natural killer (NK) cells, monocytes, macrophages, and dendritic cells are the main players in the non-specific first-line innate immune response. They combat pathogens and tumor cells by secreting cytokines such as interleukins (IL), interferons (IFN), tumor necrosis factors (TNF) and are also involved in the induction of inflammation and a more specific adaptive immune response by attracting immune cells to the tumor microenvironment [11].

NK cells play a pivotal role in the non-specific immune response and tumor surveillance by recognizing the loss or downregulation of major histocompatibility complex class I (MHC-I) molecules. In healthy cells, MHC-I expression inhibits the cytotoxic activity of NK cells by engaging inhibitory receptors. However, during oncogenic transformation, tumor cells often downregulate or lose MHC-I expression, rendering them susceptible to NK cell-mediated programmed cell death. Consequently, NK cells have emerged as promising therapeutic tools with significant antitumor potential [12,13].

**Figure 2:**
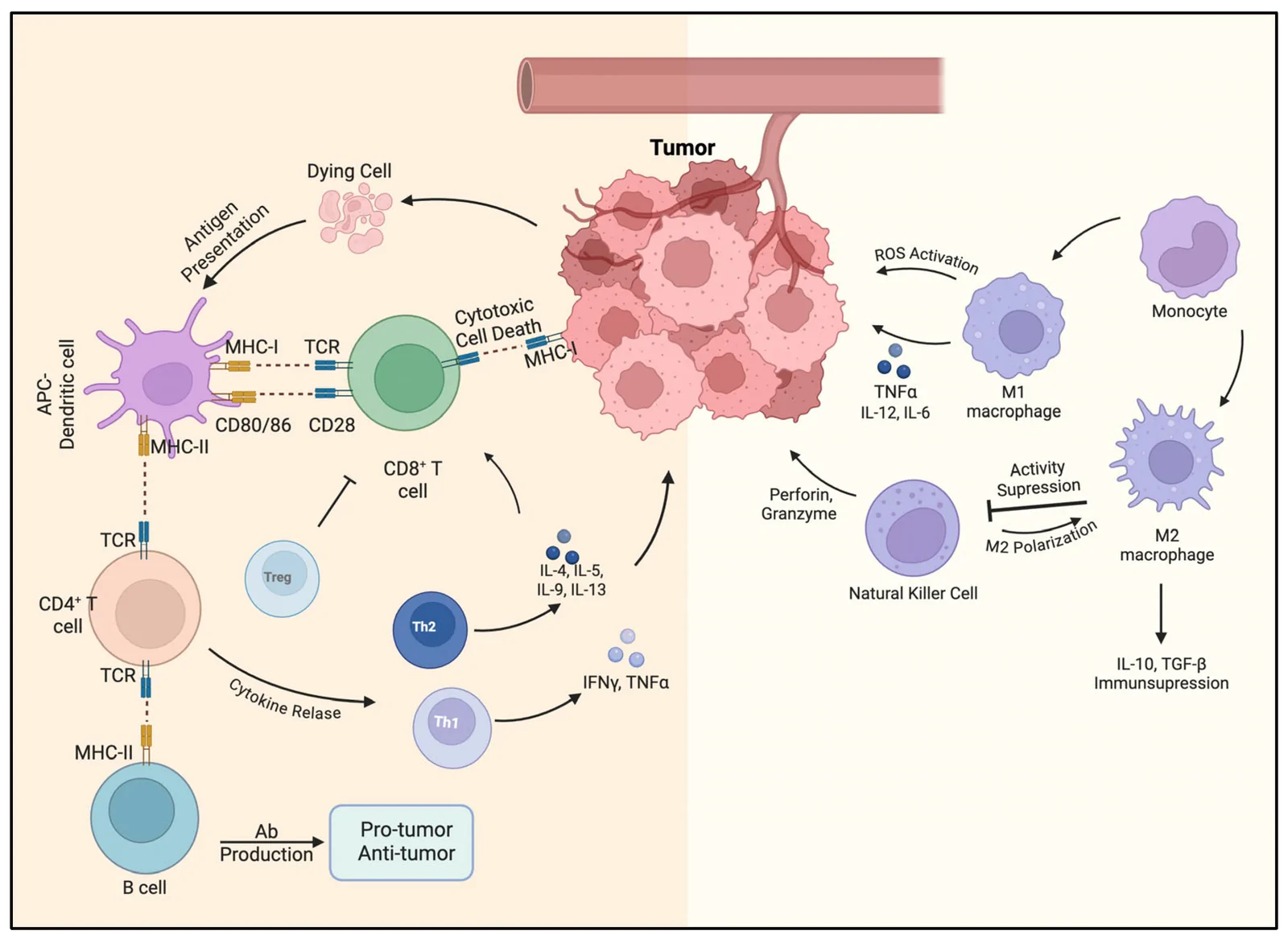
Schematic illustration of adaptive and innate immune cell interactions in the tumor microenvironment. T cells, B cells, macrophages, dendritic cells, and NK cells interact dynamically, with their activation, differentiation, and function frequently modulated by miRNAs. Created in BioRender. Biltekin, E. (2026) https://BioRender.com/d0a1033 (accessed on 29 June 2026).

Macrophages are derived from monocytes and may function as either inflammatory/anti-tumor or pro-tumorigenic cells by promoting the progression and metastatic phases of tumorigenesis. While M1 macrophages exhibit inflammatory and anti-tumorigenic functions, M2 macrophages or TAMs promote angiogenesis, tumor cell proliferation, drug resistance, invasion and metastasis [14]. Activation of adaptive immunity is mediated by the antigen presentation function of macrophages and one of the critical steps in the development of a complete immune response is the initiation of adaptive and antigen-specific T-cell activation. In addition to macrophages, dendritic cells (DCs) are major antigen-presenting cells that orchestrate and activate tumor antigen-specific T-cell responses [15,16] (Figure 2).

miRNAs have been shown to be key regulators of the maturation, differentiation and polarization of immune effector cells [17,18,19,20] (Table 1). Sullivan et al. showed that tumor suppressor miRNAs miR-15 and miR-16 control NK cell maturation by targeting the transcription factor Myb [21]. In another study, Ghani, S. et al. showed that transplantation of miR-146a overexpressing hematopoietic stem cells into mice demonstrated a role for miR-146a in monocytic differentiation in the peritoneal cavity [22]. In addition, miR-223 influenced macrophage polarization; in particular, it was shown to favor the activation of M2 macrophages in adipose tissues [23,24]. Similarly, miR-21 is involved in macrophage polarization by regulating STAT3 and its deficiency promotes the M2 phenotype [25]. Moreover, miR-17-5p mimic treatment of immature dendritic cells inhibits the transition of lipopolysaccharide-induced maturation in gastric cancer [26]. Overall, miRNAs engage in the regulation of innate immune cells and their maturation, polarization and functional plasticity in the context of cancer. However, most of these findings are derived from in vitro or mouse models, with limited clinical validation across heterogeneous cancer types.

#### 2.1.2. Regulation of Inflammatory Signaling

Beyond cellular differentiation, miRNAs also modulate pathogen recognition and downstream inflammatory signaling, including Toll-like receptors (TLRs). TLRs that are in the cell membrane or the endosomal membrane are responsible for recognizing pathogen-associated molecular patterns to maintain homeostasis; their activation may trigger NF-kB, MAPK, or IL signaling. In addition to the roles in tumor cell recognition and tumor elimination mechanisms mediated through activation of dendritic cells, macrophages and T cells, miRNAs may promote inflammation and the progression of tumor cell proliferation, angiogenesis and metastasis [27].

miR-29 has been shown to exert a pro-inflammatory role in dendritic cells by binding to endosomal TLRs, thereby activating NF-kB signaling and promoting TNF-α secretion and/or inducing apoptosis [28,29]. Similarly, exosomes carrying miR-21 and miR-29 were shown to bind endosomal TLRs of macrophages and activate TNF-α and IL-6 secretion through NF-kB signaling in lung cancer cells. Moreover, the anti-miR-21 and anti-miR-29 combinatorial therapy led to the suppression of in vivo tumor growth [30].

#### 2.1.3. Antigen Presentation

Alterations in tumor-associated antigen expression or the antigen presentation process are tightly associated with tumor immune escape and are common during tumorigenesis [31,32]. miRNAs have important regulatory roles in antigen presentation and recognition. Gao et al. showed the regulatory role of miR-9 on MHC I molecule expression and IL signaling in nasopharyngeal carcinoma cells. They reported a positive correlation between miR-9 expression and MHC-I expression [33]. In esophageal carcinoma, miR-125a-5p and miR-148a-3p regulate MHC-I expression and their expression is negatively correlated with patient survival, potentially by impairing immune-mediated tumor surveillance [34]. In another study, Colangelo et al. showed that miR-27a is highly expressed in colorectal cancer and knockdown of miR-27a led to an increase in MHC-I expression while anti-miR-27a treatment led to suppression of in vivo tumor growth [35].

### 2.2. Regulation of the Adaptive Immune Response by miRNAs

T cells and B cells are key components of antigen-specific adaptive immunity. Activation of adaptive immunity begins with the activation of naïve T cells and T-cell receptor (TCR) signaling which is initiated by either antigen presenting cells including DCs, macrophages or CD4^+^ T cells or through cytokine secretion; CD4^+^ T cells boost CD8^+^ T-cell proliferation and B-cell differentiation (Figure 2). While CD8^+^ T cells initiate cytotoxic cell death by directly binding cancer cells, B cells may differentiate into antibody secreting plasma cells and produce antibodies (Immunoglobulins/Igs) or home to bone marrow where they remain as memory cells. In addition to two main subgroups of T cells—1) CD4^+^ activated by MHC Class II dependent and 2) CD8^+^ activated by MHC Class I—a small fraction of CD4^+^ T cells called Treg cells regulate the immune system and maintain homeostasis by suppressing autoimmunity [11,36].

#### 2.2.1. T-Cell Differentiation and Activation

miRNAs exert profound effects on T-cell development and biology. miR-181a is a crucial miRNA in the thymus that acts as a molecular fine tuner of TCR signaling by repressing multiple phosphatases, lowering the threshold of T-cell activation. Li et al. demonstrated that miR-181a is more highly expressed in CD4 and CD8 double-positive cells than in mature T cells. When they inhibited miR-181a by using antagomiR, they observed decreased levels of CD4^+^ single positive cells in thymocytes highlighting the role of miR-181a in T-cell maturation. Also, overexpression of miR-181a caused T-cell activation due to higher IL-2 levels and TCR signaling [37]. miR-181a is also overexpressed in T_H_17 cells and mediates its effect through phosphorylation of ERK and its inhibition suppresses the activation of T_H_17 cells and cell proliferation [38]. Moreover, the miR-17-92 cluster induces T-cell proliferation. Separately, miR-29 is involved in T-cell differentiation through negative regulation of IFN-γ [39]. Skinner et al. showed that the knockdown of the miR-17-92a cluster reduces the number of Tregs with age and causes induction of apoptosis and suppression of proliferation of Treg cells [40].

In contrast to its predominantly oncogenic role in non-small cell lung cancer, miR-150 exerts crucial functions in CD8^+^ T cells and promotes effector CD8^+^ T-cell expansion, differentiation, and cytolytic capacity, including granzyme B expression. However, miR-150 also negatively regulates the formation of long-lived memory CD8^+^ T cells [41,42,43]. Also, He et al. showed the anti-tumorigenic potential of miR-21. They showed that miR-21-deficient mice exhibit a lower number of CD4^+^ and CD8^+^ T cells in correlation with low IFN-γ and IL-2 levels and enhanced tumor sizes [44]. Additionally, miR-155 acts as an epigenetic regulator of T-cell differentiation by regulating PRC2 and its subunit Ezh2 [45]. In addition, high Ezh2 CD8^+^ T cells have been associated with longer patient survival in ovarian cancer (OC) patients and miR-101 and miR-26a promoted apoptosis in the CD8^+^ T cell group while suppressing Ezh2 expression highlighting its role in cancer immunity [46].

#### 2.2.2. B-Cell Differentiation and Activation

B cells exert their effects either by presenting antigens for T cells, secreting antibodies (Igs), or cytokines like IL-10, which may regulate the CD8^+^ T cell, TNF-α and IFN-γ secretion. While their antigen-presenting role may trigger cytotoxic T-cell activity, their immune regulatory role is exhibited through a subset of B cells (B regs), which release IL-10, IL-35, and TGF-β. B regs may function as immunosuppressors inducing pro-tumorigenic cytokines [47,48]. In addition to its regulatory role in T-cell differentiation, miR-155 also plays a role in the development of B-cell lymphomas. In one of the very early studies, Croce et al. compared miR-155 expression in mice B220^+^/IgM^−^ pre-B cells and B220^+^/IgM^+^ mature B cells and their findings showed that miR-155 was 10-fold overexpressed in pre-B cells and knockdown of SHIP, the target of miR-155, leads to suppression of B-cell differentiation [49]. In another study, B-cell lymphomas (Chronic Lymphocytic Leukemia, Diffuse Large B-Cell Lymphoma, Burkitt Lymphoma, Follicular Lymphoma) were analyzed and downregulation of miR-28 was linked to these malignancies; administration of miR-28 suppressed B-cell receptor signaling, Lymphoma B cells (%) and slowed Ramos BL cell xenograft tumor growth [50].

**Table 1:** miRNAs regulating key immune cells in the tumor microenvironment.

Selected Immune Cells	miRNAs	Effect on the Cell (from the Text)	References
**NK cells**	miR-15, miR-16	Regulate NK cell maturation through the transcription factor Myb	[21]
**Monocytes**	miR-146a	Involved in monocytic differentiation	[22]
**Macrophages (M1/M2/TAMs)**	miR-223	Favors activation of M2 macrophages	[23,24]
	miR-21	Involved in macrophage polarization by regulating STAT3; its deficiency promotes M2 phenotype	[25]
	Exosomal miR-21, miR-29	Bind endosomal TLRs of macrophages and activate TNF-α and IL-6 secretion through NF-κB signaling	[30]
	Exosomal miR-222-3p	Induces M2-like TAM states through suppression of SOCS3 and induction of STAT3 signaling	[51]
**Dendritic cells (DCs)**	miR-17-5p	Inhibits the transition of LPS-induced maturation of immature dendritic cells	[26]
	miR-29	Exerts a pro-inflammatory role by binding the endosomal TLRs, activating NF-κB signaling and promoting TNF-α secretion	[28,29]
**T cells (CD4^+^/CD8^+^/** **Treg/Th17)**	miR-181a	Highly expressed in double-positive thymocytes; regulates T-cell maturation and TCR signaling threshold; overexpressed in Th17 cells and promotes their activation via ERK	[37,38]
	miR-17-92 cluster, miR-29	Induces T-cell proliferation; miR-29 involved in T-cell differentiation through negative regulation of IFN-γ; knockdown of miR-17-92a reduces Treg numbers and induces apoptosis	[39,40]
	miR-150	Promotes effector CD8^+^ T-cell expansion, differentiation and cytolytic capacity (including granzyme B); negatively regulates long-lived memory CD8^+^ T cells	[41,42,43]
	miR-21	Deficiency leads to lower numbers of CD4^+^ and CD8^+^ T cells, reduced IFN-γ and IL-2, and enhanced tumor size	[44]
	miR-155	Acts as an epigenetic regulator of T-cell differentiation through PRC2 and its subunit Ezh2	[45]
	miR-101, miR-26a	Promote apoptosis in CD8^+^ T cells while suppressing Ezh2 expression	[46]
**B cells**	miR-155	Overexpressed in pre-B-cells; knockdown of its target SHIP suppresses B cell differentiation	[49]
	miR-28	Downregulation linked to B-cell lymphomas; administration suppresses BCR signaling and slows lymphoma growth	[50]

### 2.3. Regulation of Immune Checkpoint Molecules by miRNAs

Immune homeostasis is controlled through immune checkpoint molecules including CTLA-4, PD-1, PD-L1, LAG3, and TIM3. They either promote or suppress the activation of T cells and are considered the master controllers of immune responses [52,53].

#### 2.3.1. CTLA-4 and CD28 Regulation by miRNAs

CTLA-4 (Cytotoxic T-lymphocyte-associated protein 4) is the first reported immune checkpoint protein that suppresses immune activation, and its inhibition represents the first implication of immunotherapy in melanoma patients [54]. CTLA-4 is expressed on the surface of T cells and exerts vital suppressor activity on T cells through the ligands CD80 and CD86, which are members of the B7 family and are located on antigen-presenting cells (APCs) [55,56,57]. On the other hand, engagement of the costimulatory CD28 molecule with CD80/CD86 acts in contrast to CTLA-4, in addition to TCR and MHC induce T-cell activation. CD28 promotes proliferation and secretory activities of T cells [58].

Vaddi et al. identified miR-155 as a context-dependent regulator of CTLA-4 in metastatic melanoma patients and showed that it suppresses CTLA-4 mRNA levels in Treg cells and is associated with poor prognosis in metastatic melanoma patients. Inhibition of miR-155 can be used to modulate CTLA-4 levels, suggesting a strategy for immunotherapeutic approaches in melanoma patients [59]. Richardsen et al. found that decreased expression of miR-424-3p in prostate cancer is associated with poor patient survival. They also reported a negative association between CTLA-4 and miR-424-3p and suggest a potential miR-424/CTLA-4 axis as a therapeutic target in prostate cancer [60]. Moreover, in non-small cell lung cancer, researchers reported a significant decrease in expressions of miR-155 and miR-630. Using prediction algorithms identified that these two miRs have direct binding sites in CTLA-4 and suggest their role in regulating immune response [61].

In contrast to the inhibitory activity of CTLA-4, CD28 shows co-stimulatory effects on T cells, regulates the PI3K, NF-kB, and AKT-mTOR pathways, and controls the proliferative capability and secretory functions of T cells [62,63]. Klein Geltink et al. revealed that CD28 signaling temporarily suppresses miR-33, thereby increasing expression of carnitine palmitoyl-transferase 1a (CPT1a), a rate-limiting enzyme in fatty acid oxidation. This metabolic reprogramming supports the generation of memory T cells and enhances anti-tumor immunity [64].

#### 2.3.2. Regulation of PD-1 and PD-L1 by miRNAs

PD-1/PD-L1 signaling is the most extensively studied key axis in immune-checkpoint targeted therapies. PD-1 (PDCD1/CD279) is a transmembrane protein and a member of the CD28 family, located on a heterogeneous population of innate immune cells including natural killer cells, macrophages, dendritic cells, monocytes, and myeloid-lineage cells, as well as adaptive immune cells including activated T and B lymphocytes [65,66]. PD-1 shares 20% homology with the CTLA-4 amino acid sequence and plays an inhibitory role in the proliferation and activation of T cells [67].

miRNAs play a crucial role in regulating PD-1 expression on T cells (Table 2). Notably, miR-138-5p directly targets the 3′UTR of PD-1 mRNA, reduces its surface expression on DCs and T-cells, and reverses T cell exhaustion in lung cancer models [68]. Similarly, Zhang et al. demonstrated that miR-149-3p modulates PD-1 along with other inhibitory receptors such as TIM-3 and BTLA; thus, it enhances T-cell activation and anti-tumor immunity [69]. These regulatory mechanisms highlight the therapeutic potential of miRNA-based approaches to potentiate immune checkpoint blockade.

PD-L1 (B7-H1, Programmed cell death 1 ligand, CD274) is the ligand for PD-1 and is expressed on tumor cells, various innate immune cells, macrophages (M2s), DCs, MDSCs, B regs and NK cells. The interaction between PD-1 and PD-L1 is one of the key mechanisms of immune suppression, maintenance of immune homeostasis, and protection of cells from undesirable immune eradication. Therefore, PD-L1 expression on tumor cells provides an escape mechanism from immune detection by reducing T-cell activity and further eradication of tumor cells [70].

Several studies have revealed the direct interaction of miRNAs with PD-L1 mRNA (Table 2). One of the earliest studies in biliary epithelial cells revealed a direct binding between miR-513 and PD-L1 mRNA, and transfection of antisense miR-513 induced PD-L1/B7-H1 protein expression, while precursor miR-513 suppressed IFN-γ-induced PD-L1 expression and apoptotic cell death [71]. The miR-200 family has also been shown to bind directly to PD-L1 mRNA in several cancer models including lung cancer, OC, breast cancer, and colorectal cancer. Seminal work by Chen et al. demonstrated that miR-200 family members (particularly miR-200b and miR-200c) directly target the 3′UTR of PD-L1; therefore, they link the epithelial–mesenchymal transition (EMT) program regulated by ZEB1 to intratumoral immunosuppression and metastasis [72]. This axis enables tumor cells to evade CD8^+^ T-cell-mediated cytotoxicity while low miR-200 levels correlate with elevated PD-L1 expression and poor response to immune checkpoint inhibitors (ICIs).

Subsequent studies further expanded this regulatory network and several groups showed that miR-34a, induced by p53, directly binds to the PD-L1 3′UTR, suppressing surface PD-L1 expression, reducing PI3K/AKT signaling, and enhancing CD8^+^ tumor-infiltrating lymphocytes with increased IFN-γ and TNF-α production in non-small cell lung cancer (NSCLC) and triple-negative breast cancer (TNBC) models [73,74,75]. Similarly, miR-138-5p and miR-424 (miR-15/16 family) directly target the PD-L1 gene, reverse T-cell exhaustion, and synergize with chemotherapy or ICI therapies in lung and OC [76,77]. Other miRNAs such as miR-4458, miR-873, and miR-320a also contribute to PD-L1 downregulation across multiple solid tumors and highlight miRNAs as a versatile post-transcriptional layer that controls the PD-1/PD-L1 immune checkpoint axis [78,79,80]. However, PD-L1 regulation by miRNAs is a complex and multilayered process and PD-L1 expression involves both post-transcriptional (miRNA) and transcriptional control. While most reported miRNAs suppress PD-L1, studies show context-dependent upregulation in specific cancer types or microenvironments [81].

**Table 2:** Selected miRNAs regulating PD-1/PD-L1 in cancer.

miRNA	Type	Target	Cancer Types	Main Effect on PD-1/PD-L1	References
miR-34a	Mimic	PD-L1 (3′UTR)	NSCLC, TNBC, OC	Directly binds PD-L1 3′UTR, suppresses surface PD-L1 expression, reduces PI3K/AKT signaling, enhances CD8^+^ TILs with increased IFN-γ and TNF-α	[73,75,82]
miR-200 family	Mimic	PD-L1 (3′UTR)	Lung, Breast, Ovarian	Directly targets PD-L1 3′UTR, links EMT via ZEB1 to immunosuppression, enables evasion of CD8^+^ T-cell cytotoxicity	[76,81,83,84]
miR-149-3p	Mimic	PD-1, TIM-3, BTLA	Breast Cancer	Reduces PD-1 (and other inhibitory receptors), enhances T-cell activation and anti-tumor immunity	[69]
miR-138-5p	Mimic	PD-L1 and PD-1	Glioma, Lung cancer	Directly targets PD-1 3′UTR on T cells and PD-L1 on tumor cells, reverses T-cell exhaustion	[68,76,85]
miR-424 (miR-15/16 family)	Mimic	PD-L1	Ovarian, HCC	Directly targets PD-L1, reverses T-cell exhaustion, synergizes with chemotherapy or ICI	[77,86]
miR-873	Mimic	PD-L1	Breast cancer	Directly binds PD-L1, downregulates PD-L1, reduces stemness and chemoresistance	[80]
miR-155	Anti-miR	Indirect (via signaling)	Lymphoma, Solid tumors	Context-dependent regulation of PD-L1	[87]
miR-21	Anti-miR	Indirect	Breast cancer	Reduces M2 TAMs and decreases PD-L1 via NF-κB pathway	[88,89]

#### 2.3.3. Regulation of LAG-3 by miRNAs

LAG-3 (Lymphocyte activation gene-3, also known as CD223) is an emerging immune checkpoint receptor that negatively regulates T-cell activation, proliferation, and effector functions, plays a critical role in maintaining immune homeostasis, and contributes to tumor immune evasion [90]. LAG-3 is expressed on activated CD4^+^ and CD8^+^ T cells, Tregs, NK cells and some dendritic cells and binds primarily to MHC class II molecules with higher affinity than CD4 but also interacts with other ligands such as fibrinogen-like protein 1 (FGL1), galectin-3, and LSECtin [91].

Similar to PD-1, LAG-3 is frequently co-expressed with other inhibitory receptors such as PD-1 and TIM-3 on exhausted tumor-infiltrating lymphocytes (TILs) [92,93]. Several studies have elucidated the regulation of LAG-3 by miRNAs, although this field is less mature compared to the PD-1/PD-L1 signaling axis. Recently, Yang et al. found that the miR-15/16 family modulates LAG-3 expression indirectly through the mTOR pathway. Deficiency in miR-15/16 leads to downregulation of LAG-3, along with PD-1 and TIM-3, on glioma-infiltrating CD8^+^ T-cells, reduced T-cell exhaustion, and enhanced anti-tumor immunity in mouse glioma models [94]. Additionally, miRNAs, including miR-21-5p, have been reported to elevate LAG-3 expression in certain contexts such as head and neck cancer (HNSCC) [95].

Overall, miRNA-mediated regulation of LAG-3 is highly context-dependent and varies by tumor type. Similar to PD-L1, the regulatory network for LAG-3 is complex. While most studies focus on indirect effects through signaling pathways, direct 3′UTR targeting of LAG-3 by miRNAs remains less characterized in the context of tumor immunology. Further research is required to clarify in vivo relevance, variances across cancer types and the therapeutic potential of combining miRNA modulation with LAG-3 blockade.

#### 2.3.4. Regulation of TIM-3 by miRNAs

TIM-3 (T-cell immunoglobulin and mucin domain-containing protein 3) is one of the key inhibitory immune checkpoint receptors that negatively regulate T-cell activation, proliferation and effector functions. It is expressed on activated CD4^+^ and CD8^+^ T cells, regulatory T cells (Tregs), NK cells, dendritic cells, and macrophages, where it mainly exerts an inhibitory effect on their function. TIM-3 binds to multiple ligands including galectin-9 (Gal-9), phosphatidylserine (PtdSer), high-mobility group box 1 (HMGB1) and CEACAM where it shows its function on T-cell exhaustion and suppression of anti-tumor immunity, particularly in chronic inflammatory and tumor microenvironments [96]. Like PD-1 and LAG-3, TIM-3 is frequently co-expressed with other inhibitory receptors on exhausted TILs, and dual or triple blockade strategies such as TIM-3 + PD-1 have shown promising synergistic effects in preclinical models [97,98].

Regulation of TIM-3 by miRNAs is an emerging area, although less extensively studied than the PD-1/PD-L1 axis [99]. Several miRNAs directly or indirectly target TIM-3. miR-498 directly suppresses TIM-3 expression in acute myeloid leukemia (AML) cell lines and leads to reduced cell proliferation and increased apoptosis [100]. miR-149-3p has been shown to bind the 3′UTR of TIM-3 as well as PD-1 and BTLA. miR-149-3p mimic treatment limited tumor immune escape and promoted T-cell activation in breast cancer models, highlighting its potential role in overcoming T-cell exhaustion [69]. Additionally, miR-155 exhibits complex regulatory effects on TIM-3 expression. In colon cancer cells, increased TIM-3 levels correlated with decreased miR-155 expression, promoting M2 macrophage phenotype polarization and tumor progression [101]. The miR-15/16 family also indirectly influences TIM-3 levels through the mTOR pathway in glioma models [94].

However, similar to other checkpoints, miRNA regulation of TIM-3 involves complex and context-specific mechanisms. While most validated miRNAs act as negative regulators, the therapeutic potential of miRNA mimics or inhibitors to enhance TIM-3 blockade remains to be fully explored in vivo and in clinical settings. Further studies are needed to clarify direct targeting mechanisms, cancer-type specificity, and synergy with existing immune checkpoint inhibitors.

#### 2.3.5. Regulation of CD28 and CD80/86 by miRNAs

CD28 is the primary co-stimulatory receptor on T cells that binds to CD80 and CD86 ligands on antigen-presenting cells. This interaction delivers the essential second signal, in addition to TCR-MHC interaction, for full T-cell activation, proliferation, cytokine secretion, and survival through activation of key pathways such as PI3K/AKT/mTOR and NF-κB [102,103].

CD28 functions by amplifying TCR/CD3 complex signaling. It enhances Lck and ZAP-70 activation at the immunological synapse, strengthens the magnitude and duration of the primary TCR signal, and therefore promotes full T-cell activation, proliferation, and effector function [104,105,106].

Several miRNAs modulate CD28 expression and signaling, either directly or indirectly. miR-24-3p and miR-27a-3p directly target the 3′UTR of CD28 and contribute to the age-related decline in CD28 expression on T cells. In the context of cancer and chronic stimulation, these miRNAs can dampen co-stimulatory signaling [107]. Conversely, miR-181a has been shown to increase CD28 levels while decreasing the inhibitory receptor CTLA-4 [37,108].

A key metabolic link was revealed by Klein Geltink et al., who demonstrated that CD28 co-stimulation transiently suppresses miR-33, which leads to the upregulation of CPT1a and enhanced fatty acid oxidation. This reprogramming supports memory T-cell formation and sustains anti-tumor immunity [64].

miRNAs also regulate the ligands CD80 and CD86 on antigen-presenting cells and tumor cells; therefore, they modulate the strength of co-stimulation [109]. For example, miR-424 targets both CD28 and CD80, impairs co-stimulatory signaling in colorectal cancer and contributes to resistance against immune checkpoint inhibitors [110]. miR-134 has been shown to directly target CD86 (B7-2) in melanoma by reducing its expression and promoting tumor immune escape [111]. Other miRNAs, such as miR-145, can indirectly affect the CD28-CD80/86 axis [112].

Overall, miRNA-mediated regulation of the CD28 co-stimulatory pathway and its ligands represents an important layer in fine-tuning T-cell activation versus inhibition. Similar to inhibitory checkpoints, these regulatory networks are highly context-dependent (e.g., aging, cancer type and tumor microenvironment). Therapeutic modulation of these miRNAs holds potential to enhance co-stimulation and synergize with existing immunotherapies.

### 2.4. Exosomal miRNAs in Tumor–Immune Crosstalk

Exosomes are extracellular vesicles smaller than 150 nm and serve as critical mediators of intercellular communication within the TME [113]. Tumor-derived exosomes enable cancer cells to deliver specific miRNAs that reprogram immune cell phenotypes and promote immune evasion [114]. For example, in HNSCC, these vesicles have been shown to alter LAG-3 expression on infiltrating immune cells and they contribute to T-cell exhaustion and local immune suppression [115]. Similarly, exosomes carrying miR-21 and miR-29 were shown to bind endosomal TLRs of macrophages and activate TNF-α and IL-6 secretion through NF-kB signaling in lung cancer cells. Moreover, the anti-miR-21 and anti-miR-29 combinatorial therapy led to suppression of in vivo tumor growth [30]. In another study, Ying et al. found that miR-222-3p was enriched in epithelial OC exosomes and that derived exosomes induced M2-like TAM states through suppression of SOCS3 and induction of STAT3 signaling in an in vitro model [51]. These observations highlight the dual capacity of tumor-derived exosomal miRNAs to shape inhibitory receptor expression such as LAG-3, and to amplify inflammatory pathways that reinforce immune escape.

Strategies that aim to neutralize key immunosuppressive exosomal miRNAs through anti-miR oligonucleotides or to engineer therapeutic miRNA-loaded exosomes may benefit current approaches [116,117,118]. Such interventions may enhance the efficacy of therapies, including immune checkpoint inhibitors and chemotherapy, and may position exosome-targeted strategies as an important component of next-generation cancer immunotherapy.

## 3. Therapeutic Potential of miRNAs in Cancer Immunotherapy and Future Directions

miRNAs can simultaneously regulate multiple gene expressions and related pathways within cancer and immune cells in the TME [119,120,121]. Thus, they are attractive therapeutic agents for reprogramming the tumor-immune crosstalk, either alone or in combination with other treatment modalities, including chemotherapy, immune checkpoint inhibitors, and targeted therapies. Both miRNA mimics for restoring tumor-suppressive miRNAs and anti-miRs for inhibiting oncogenic miRNAs have shown promise in preclinical models and early clinical trials [122].

### 3.1. Combination Strategies with Current Treatment Modalities: Chemotherapy, Immune Checkpoint Inhibitors (ICIs) and Targeted Therapeutics

#### 3.1.1. Combination Strategies with Current Treatment Modalities: Immune Checkpoint Inhibitors (ICIs)

Combining miRNA therapeutics with immune checkpoint inhibitors (ICIs) may represent a promising strategy in cancer immunotherapy. By simultaneously targeting multiple immune-regulatory pathways, miRNA-based agents can potentiate the efficacy of anti-PD-1/PD-L1 and anti-CTLA-4 therapies and help overcome primary and acquired resistance.

Preclinical studies have demonstrated strong synergy with several miRNA candidates. Kim et al. showed that anti-miR-21 reduces M2-like macrophage polarization in a mouse melanoma model and suppresses RAS/MEK/ERK and PTEN/AKT/mTOR signaling pathways that sensitize tumors to PD-1/PD-L1 inhibitors in mouse colorectal cancer, breast cancer, and lung metastasis models [89,123]. miR-424-5p mimics have been shown to downregulate PD-L1 expression and enhance pro-inflammatory macrophage polarization, which results in improved anti-tumor immunity when combined with PD-1 blockade [86]. Such miRNA-ICI combinations hold significant potential to broaden the patient population that responds to checkpoint inhibitor-based therapies.

#### 3.1.2. Combination Strategies with Chemotherapy

Chemoresistance remains a major obstacle in successful cancer treatment, and it is closely intertwined with immune evasion. Recent studies have shown that immune checkpoint/chemotherapy resistance is frequently regulated by miRNA-checkpoint axes. Gao et al. showed that miR-873 directly binds to PD-L1 and the miR-873/PD-L1 axis regulates breast cancer stemness, immune evasion and doxorubicin-based chemoresistance through PI3K/ERK/AKT signaling in in vivo models [80].

Li et al. reported downregulated levels of miR-766-5p in carboplatin resistant OC cells. While overexpression of miR-766-5p demonstrated increased carboplatin sensitivity, they also validated PD-L1 as a direct target of miR-766-5p [124]. Similarly, in cisplatin-resistant lung and OC models, decreased levels of miR-526b-3p, miR-34a-5p, and miR-145 were detected, accompanied by elevated PD-L1 levels. Their restoration led to sensitization of cells to platinum-based chemotherapy and enhanced CD8^+^ T-cell activity through oncogenic signaling mechanisms, including the STAT3/PD-L1 and c-Myc/PD-L1 axes [82,125,126]. Overall, these findings highlight miRNAs as key regulators linking chemoresistance and immune suppression.

#### 3.1.3. Combination Strategies with Therapeutics

Beyond traditional chemotherapy and immune checkpoint inhibitors, miRNAs also show promising synergistic potential when combined with targeted therapies such as PARP inhibitors and cetuximab. PARP inhibitors, particularly olaparib, have been shown to upregulate PD-L1 expression in hepatocellular carcinoma by suppressing miR-513 [127]. This mechanism creates a strong rationale for combining PARP inhibition with anti-PD-1 therapy, as the dual approach can simultaneously exploit DNA repair deficiency and enhance anti-tumor immune responses.

In HER2-positive breast cancer and colorectal cancer, trastuzumab/cetuximab-based regimens can be potentiated by miRNA-based therapies and target modulation. For example, the circ_0001598/miR-1184/PD-L1 axis contributes to trastuzumab resistance, while miRNA restoration (e.g., miR-1184 mimics) sensitizes cells to EGFR-targeted therapy [128]. Similarly, Xu et al. reported a negative correlation between PD-L1 and miR-20b-5p expression, accompanied by decreased levels of miR-20b-5p in CRC patients. Inhibition of miR-20b-5p through *HCG18* resulted in sensitivity to cetuximab treatment and suppressed PD-L1 expression [129]. These combination strategies highlight the versatility of miRNA therapeutics in enhancing the effectiveness of precision oncology approaches.

### 3.2. miRNA-Mimic-Based Therapeutics and Their Clinical Implications

miRNA mimics are synthetic double-stranded RNA molecules that are designed to restore the function of downregulated miRNAs that promote tumor suppressor functions in cancer cells and the tumor microenvironment. These mimics are typically loaded into the RNA-induced silencing complex (RISC), function identically to endogenous mature miRNAs, and lead to sequence-specific repression of target mRNAs [130]. By restoring the expression of key tumor-suppressive miRNAs that are frequently lost during tumorigenesis, miR mimics can simultaneously modulate multiple oncogenic pathways, including immune checkpoint expression, effector cell function and EMT [131].

One of the most well-studied miRNA mimics in the context of cancer immunotherapy is miR-34a. As a direct transcriptional target of p53, miR-34a mimics have been shown to downregulate PD-L1 and enhance CD8^+^ T-cell infiltration [73,74,75]. Similarly, mimics of the miR-200 family (particularly miR-200b and miR-200c) reverse EMT-associated immunosuppression [72,83]. Other promising candidates such as miR-138-5p, miR-16, and miR-193a-3p mimic have demonstrated the ability to reverse T-cell exhaustion, modulate macrophage polarization and improve anti-tumor immunity in various preclinical models [85,132,133]. Some of these mimics (e.g., MRX34 and TargomiR) have advanced into clinical trials, although with mixed results [134].

Despite their strong mechanistic rationale and preclinical efficacy, the clinical translation of miRNA mimics has faced notable challenges. MRX34 (liposomal miR-34a mimic) was the first miRNA mimic to reach clinical trials and showed encouraging immune-modulating effects, but it was ultimately terminated in Phase 1 due to severe immune-related adverse events [135,136]. These experiences have highlighted the need for improved delivery systems and more refined patient selection strategies. Nevertheless, miRNA mimics remain highly attractive candidates for combination immunotherapy due to their ability to simultaneously target multiple nodes in the cancer-immune axis (Table 3).

**Table 3:** Selected miRNA therapeutics in clinical development with immunomodulatory relevance.

Therapeutic Agent	Type	Target miRNA	Delivery System	Cancer Indications	Phase/Status	Key Immunomodulatory Effects	Ref.
MRX34	Mimic	miR-34a	Liposomal nanoparticle (NOV40)	Solid tumors (NSCLC, HCC, melanoma)	Phase 1 (terminated due to irAEs)	Suppresses PD-L1, Induces CD8^+^ TILs, IFN-γ and TNF-α	[135]
TargomiR (MesomiR-1)	Mimic	miR-16	Bacterial minicells (EDV)	Malignant pleural mesothelioma, NSCLC	Phase 1 (completed)	Modulation of PD-L1 and immune activation	[134]
Cobomarsen (MRG-106)	Anti-miR	miR-155	LNA-modified oligonucleotide	Cutaneous T-cell lymphoma (CTCL), other lymphomas	Phase 2 (terminated for business reasons)	Suppresses Treg activity, modulation of TME	[137]
TTX-MC138	Anti-miR	miR-10b	Dextran-coated iron oxide NP	Metastatic breast cancer, GBM	Phase 1/2 (Phase1a completed, Phase 2 ongoing)	Modulation of metastasis and immune evasion	[138]
INT-1B3	Mimic	miR-193a-3p	Lipid nanoparticle	Advanced solid tumors	Phase 1/2 (terminated due to insufficient funding)	Immune modulation in TME	[132]

### 3.3. miRNA Inhibitor-Based Therapeutics and Their Clinical Implications

miRNA inhibitors, also known as anti-miRs or antagomiRs, are chemically modified single-stranded oligonucleotides designed to bind and inhibit overexpressed oncogenic miRNAs (oncomiRs). These molecules prevent the interaction between the mature miRNA and its target mRNAs. In doing so, they restore the expression of tumor suppressor genes that were suppressed by the oncomiRs. Common chemical modifications such as locked nucleic acids (LNA), phosphorothioate backbones and 2′-O-methyl groups enhance their stability, binding affinity and resistance to nuclease degradation [139,140].

One of the most advanced anti-miR candidates is cobomarsen (MRG-106) which is an anti-miR-155 that has progressed to Phase 2 clinical trials in cutaneous T-cell lymphoma (CTCL) and other hematologic malignancies [137]. Inhibition of miR-155 modulates T-cell differentiation, reduces regulatory T-cell activity, and alters the tumor microenvironment in favor of anti-tumor immunity [87].

Additionally, miR-10b inhibitors have gained attention in recent years due to their potential efficacy against extremely aggressive cancers such as glioblastoma (GBM) and metastatic breast cancer [141,142]. A notable candidate is TTX-MC138, a miR-10b inhibitor conjugated with advanced dextran-coated iron oxide nanoparticles which is currently under Phase 1/2 clinical trial (ClinicalTrial ID: NCT06260774) that previously showed promising tumor-targeting properties during its preclinical development [138]. Another miR-10b inhibitor (RGLS5579) was developed specifically for glioblastoma and demonstrated significant survival benefit when combined with temozolomide in orthotopic mouse models. A related clinical study (NCT01849952) has been evaluating miR-10b expression patterns as a prognostic and diagnostic marker in glioma patients [143].

Despite encouraging preclinical results, anti-miR therapeutics face similar translational hurdles as mimics, including efficient delivery to target tissues, potential off-target effects, and immune activation. Nevertheless, their ability to suppress multiple tumor-suppressor pathways and reshape the immune landscape makes them highly attractive for both monotherapy and combination strategies with immune checkpoint inhibitors (Table 3).

### 3.4. Delivery Systems and Major Obstacles

Efficient and safe delivery of miRNA mimics and inhibitors to selectively target the tumor site remains one of the most significant barriers for the clinical translation of miR-based therapeutics [144]. Unlike small-molecule inhibitors or monoclonal antibodies, miRNA therapeutics are nucleic acids that are rapidly degraded by nucleases in circulation and poorly taken up by cells when delivered alone. Systemic administration carries the risk of off-target accumulation, particularly in the liver and kidneys, which leads to potential toxicity and reduced therapeutic efficacy [145].

Several delivery platforms have been developed to overcome these challenges. Lipid nanoparticles (LNPs), polymeric nanoparticles, inorganic NPs, exosome-based vehicles, and viral vectors such as AAV are among the most widely investigated systems. Tumor-targeted approaches that use pH-sensitive nanoparticles, ligand-conjugated carriers (e.g., EGFR or folate receptors) and biomaterial-based scaffolds have shown improved specificity in preclinical models [146,147,148,149,150]. Notably, bacterial minicells, as used in TargomiR, and advanced dextran-coated iron oxide nanoparticles, such as TTX-MC138 for miR-10b, represent innovative strategies by combining delivery with imaging capabilities [134,138,151,152].

Despite these advances, major obstacles persist. These include immunogenicity, cytokine release syndromes (as observed in the MRX34 trial), manufacturing scalability under Good Manufacturing Practice (GMP) conditions and achieving sufficient therapeutic index in humans. Off-target effects and unintended immune activation may limit the clinical application [122,135]. Addressing these challenges through next-generation chemical modifications, smart delivery systems, and biomarker-guided patient selection will be essential for the successful clinical translation and realization of the full clinical potential of miRNA-based therapeutics in cancer immunotherapy.

## 4. Spatial Transcriptomics of miRNA-Immune Interactions in the Tumor Microenvironment

The integration of spatial transcriptomics with miRNA profiling has the potential to transform our understanding of the tumor-immune cell interaction in the TME by preserving the architectural context of gene expression [153]. Unlike bulk or single-cell RNA sequencing, spatial technologies reveal highly localized miRNA and cell-type-dependent immune regulation that drives tumor immune evasion or response. Recent studies using 10x Visium, GeoMx DSP, MERFISH and Stereo-seq have shown that miRNA activity is not uniform but displays distinct spatial patterns [154,155]. For example, Su et al. constructed a prognostic risk model based on four RNA methylation-related miRNAs including miR-551a, miR-4739, miR-326 and miR-210-3p in HCC. Furthermore, they showed the spatial distribution of these miRNA risk scores within the tumor microenvironment and their association with specific immune cell subtypes. In particular, miR-4739 was found to be a regulator of SPP1+ macrophages [155]. When applied to nine different cancer types, StmiR, a novel XGBoost-based framework designed to predict spatially resolved miRNA activity from spatial transcriptomics data, identified conserved miRNA regulatory hubs such as miR-21, let-7a, miR-30 family that consistently ranked among the top active miRNAs across malignancies [156]. It also uncovered cell-type-specific miRNA signatures in fibroblasts, B cells, malignant cells and macrophages. These results highlighted the context-dependent nature of miRNA regulation within the tumor microenvironment. For instance, in malignant cells, the miR-200 family showed high predicted activity associated with EMT pathways, while in fibroblasts, the miR-30 family was linked to stromal remodeling. As spatial multi-omics approaches continue to advance, they are expected to guide precision miRNA therapeutics by identifying “miRNA-checkpoint hotspots” within the TME [156]. Another computational tool that predicts miRNA activity from single-cell RNA-seq and spatial transcriptomics data is miTEA-HiRes. It creates activity maps across cells and tissues and identifies miRNAs that show different activity between conditions. For example, several miRNAs such as miR-122-5p, miR-665, miR-125b-5p, and miR-4257 were found to be more active in migratory breast cancer cells compared to static cells [157]. Overall, this emerging field holds great promise to overcome resistance and optimization of combination strategies in cancer immunotherapy. However, it remains in its early stages with limited validation across diverse cancer types and needs standardized computational frameworks before clinical translation can be reliably achieved.

## 5. Limitations and Challenges

In vitro and in vivo preclinical findings on miRNAs have demonstrated robust and highly promising results. Moreover, miRNA-based therapeutics are currently being evaluated in clinical trials and some of them have advanced to Phase II clinical trials [144]. However, translation of miRNA-based approaches in cancer immunotherapy may require additional time due to several limitations. Most current evidence is derived from in vitro and preclinical mouse models with limited validation in heterogeneous patient populations. The context-dependent nature of miRNA functions, such as cancer type, disease stage, and tumor microenvironment, complicates generalization of findings [88,158]. Technical challenges such as potential off-target effects due to the broad regulatory networks of miRNAs, immunogenicity and inefficient delivery to the tumor site represent major challenges that must be overcome. Additionally, scalable manufacturing under GMP conditions, long-term safety profiles and potential exacerbation of immune-related adverse events require careful evaluation [130,158]. Patient heterogeneity, including differences in immune profiles and tumor mutational burden, further complicates biomarker development and personalized treatment strategies [159]. Addressing these limitations will require larger and well-designed clinical trials, integration of advanced spatial and single-cell technologies and the development of robust computational models for predicting miRNA targets.

## 6. Conclusions and Future Directions

Three decades after their discovery, miRNAs have emerged as versatile regulators of gene expression and critical cellular processes, making them potential tools for developing novel therapies for cancer or other human diseases. Recently, miRNA therapies have advanced to clinical trials showing promise as a potential targeted therapy. The role of miRNAs in cancer immunity through their influence on immune cell maturation, antigen presentation, checkpoint expression and overall tumor-immune crosstalk is an emerging field and indicates their potential use as immune-modulator therapies. Their ability to simultaneously modulate multiple targets within the same pathway offers a unique therapeutic advantage over conventional single-target agents.

While preclinical studies have demonstrated robust efficacy of miRNA mimics and inhibitors, alone or in combination with chemotherapy, ICIs, and targeted therapies, clinical translation faces significant challenges related to delivery, specificity, and safety. Nevertheless, early clinical data and biomarker studies provide encouraging results of enhanced anti-tumor immunity and improved patient outcomes.

Future research should prioritize the development of tumor-specific delivery systems, multiplex miRNA therapeutics capable of regulating multiple mRNAs rather than a single mRNA, and integration with spatial transcriptomics to map context-dependent interactions in the TME. Close collaboration between academia, industry and regulatory agencies will be critical to overcome the remaining barriers to clinical translation.

miRNAs hold transformative potential to reprogram the tumor-immune landscape and could become key components of next-generation cancer immunotherapies, offering new hope for cancer patients with limited therapeutic options.

## Data Availability

No new data were created or analyzed in this study. Data sharing is not applicable to this article.

## Abbreviations

The following abbreviations are used in this manuscript:

AAV	Adeno-Associated Virus
APCs	Antigen Presenting Cells
B-CLL	B-Cell Chronic Lymphocytic Leukemia
CD28	Cluster of Differentiation 28
CD80/CD86	B7-1/B7-2 (co-stimulatory molecules)
CTLA-4	Cytotoxic T-Lymphocyte-Associated Protein 4
DCs	Dendritic Cells
EMT	Epithelial–Mesenchymal Transition
GMP	Good Manufacturing Practice
H&E	Hematoxylin and Eosin (staining)
HNSCC	Head and Neck Squamous Cell Carcinoma
ICI/ICIs	Immune Checkpoint Inhibitor(s)
IFN-γ	Interferon Gamma
IL	Interleukin
LAG-3	Lymphocyte Activation Gene-3
LNA	Locked Nucleic Acid
MHC-I	Major Histocompatibility Complex Class I
miRNA/miRs	microRNA
mTOR	Mammalian Target of Rapamycin
NK cells	Natural Killer Cells
NSCLC	Non-Small Cell Lung Cancer
PARP	Poly (ADP-Ribose) Polymerase
PD-1	Programmed Cell Death Protein 1
PD-L1	Programmed Death-Ligand 1
R-CHOP	Rituximab + Cyclophosphamide, Doxorubicin, Vincristine, Prednisone
OC	Ovarian Cancer
RISC	RNA-Induced Silencing Complex
TAMs	Tumor-Associated Macrophages
TCR	T-Cell Receptor
TIM-3	T-Cell Immunoglobulin and Mucin Domain-Containing Protein 3
TME	Tumor Microenvironment
TNBC	Triple-Negative Breast Cancer
TNF-α	Tumor Necrosis Factor Alpha
Treg	Regulatory T cells
UMAP	Uniform Manifold Approximation and Projection
